# Glycosylation, transport, and complex formation of palmitoyl protein thioesterase 1 (PPT1) – distinct characteristics in neurons

**DOI:** 10.1186/1471-2121-8-22

**Published:** 2007-06-12

**Authors:** Annina Lyly, Carina von Schantz, Tarja Salonen, Outi Kopra, Jani Saarela, Matti Jauhiainen, Aija Kyttälä, Anu Jalanko

**Affiliations:** 1 Department of Molecular Medicine, National Public Health Institute, Biomedicum Helsinki, P.O.BOX 104, Haartmaninkatu 8, FIN-00251 Helsinki, Finland; 2 Department of Medical Genetics and Neuroscience Center, Folkhälsan Institute of Genetics, Biomedicum Helsinki, FIN-00014 University of Helsinki, Finland; 3 Department of Medical Biochemistry, Medical University of Vienna, Dr. Bohr Gasse 9, A-1030 Vienna, Austria

## Abstract

**Background:**

Neuronal ceroid lipofuscinoses (NCLs) are collectively the most common type of recessively inherited childhood encephalopathies. The most severe form of NCL, infantile neuronal ceroid lipofuscinosis (INCL), is caused by mutations in the *CLN1 *gene, resulting in a deficiency of the lysosomal enzyme, palmitoyl protein thioesterase 1 (PPT1). The deficiency of PPT1 causes a specific death of neocortical neurons by a mechanism, which is currently unclear. To understand the function of PPT1 in more detail, we have further analyzed the basic properties of the protein, especially focusing on possible differences in non-neuronal and neuronal cells.

**Results:**

Our study shows that the N-glycosylation of N197 and N232, but not N212, is essential for PPT1's activity and intracellular transport. Deglycosylation of overexpressed PPT1 produced in neurons and fibroblasts demonstrates differentially modified PPT1 in different cell types. Furthermore, antibody internalization assays showed differences in PPT1 transport when compared with a thoroughly characterized lysosomal enzyme aspartylglucosaminidase (AGA), an important observation potentially influencing therapeutic strategies. PPT1 was also demonstrated to form oligomers by size-exclusion chromatography and co-immunoprecipitation assays. Finally, the consequences of disease mutations were analyzed in the perspective of our new results, suggesting that the mutations increase both the degree of glycosylation of PPT1 and its ability to form complexes.

**Conclusion:**

Our current study describes novel properties for PPT1. We observe differences in PPT1 processing and trafficking in neuronal and non-neuronal cells, and describe for the first time the ability of PPT1 to form complexes. Understanding the basic characteristics of PPT1 is fundamental in order to clarify the molecular pathogenesis behind neurodegeneration in INCL.

## Background

Neuronal ceroid lipofuscinoses (NCLs) comprise a group of recessively inherited neurodegenerative disorders of which the infantile form, INCL, is the most severe [1]. Clinical symptoms include motor and cognitive deterioration, visual failure, and seizures, leading to death in the first or second decade of life. Pathological findings include autofluorescent lysosomal storage material, harbouring an ultrastructure of granular osmiophilic deposits (GRODs) in all tissues of the patients [2]. While most cell types remain unaffected despite the presence of storage material, cortical neurons are lost during the disease process. However, the mechanism of cell death has remained elusive.

The defective gene behind the INCL disease, *CLN1*, encodes for palmitoyl protein thioesterase 1 (PPT1) [3]. It consists of 306 amino acids, including a signal sequence of 26 amino acids and three N-linked glycosylation sites. The enzyme is transported into lysosomes of non-neuronal cells by the mannose 6-phosphate receptor (M6PR) mediated pathway [4,5]. In mouse cortical neuron cultures, PPT1 is axonally targeted and colocalizes with presynaptic markers. Furthermore, immunoelectron microscopy and cell fractionation studies have shown that neuronal PPT1 is also found in synaptosomes and synaptic vesicles [6-8] suggesting an additional function for PPT1 outside of lysosomes. *In vitro*- studies have shown that PPT1 depalmitoylates S-acylated proteins, but its native substrates have remained unknown [9]. Palmitoylation has been shown to play a critical role particularly in neurons, where active vesicular transport and intracellular signalling take place (reviewed in [10-12]).

To date, 45 disease-causing mutations have been described in the *CLN1 *gene [13]. Although the disease is classified as an infantile form of NCL, the age of onset varies depending on the mutation: nonsense and frameshift mutations always induce the classical infantile disease, whereas some missense mutations also associate with the adult-onset disease form in addition to infantile and juvenile forms [14,15]. As a result of the mutations, the activity of the PPT1 enzyme is either reduced or abolished, or the expression level of the protein is diminished [16]. The neuronal localization of PPT1 also varies depending on the disease phenotype: mutations contributing to a severe infantile disease caused the retention of the enzyme in the ER, whereas the steady state localization of the proteins carrying a juvenile-onset disease mutation was reportedly unaffected [17]. However, this observation could not be repeated in non-neuronal cells, where all the mutant polypeptides were retained in the ER. In general, the accumulation of mutant protein in the ER is not considered to affect the phenotype [18], although this has not been studied in INCL.

Even though some aspects of the glycosylation of PPT1 have been studied previously [16,19], we wanted to further analyze the effects of its three N-glycosylation sites on the activity, and especially the trafficking of PPT1, an aspect not covered previously. Contrary to earlier studies, our results show that glycosylation at N197 and N234 is essential for PPT1's activity. We also show that the same two glycosylation sites are needed for correct lysosomal targeting of PPT1. In this study, we also demonstrate that PPT1 self-oligomerizes *in vivo*. Interestingly, we show that PPT1 expressed in neurons is differentially modified when compared with non-neuronal PPT1. Furthermore, PPT1's distribution in antibody internalization assay was different when compared to a classical lysosomal enzyme AGA, suggesting that PPT1 behaves differently from the enzymes using mannose 6-phosphate pathway for their endocytosis. This study reveals new properties of the neuronal PPT1, possibly explaining the vast differences observed in the CLN1-pathogenesis in different cell types.

## Results

### The effects of N-glycosylation on the activity and transport of PPT1

**Figure 1:**
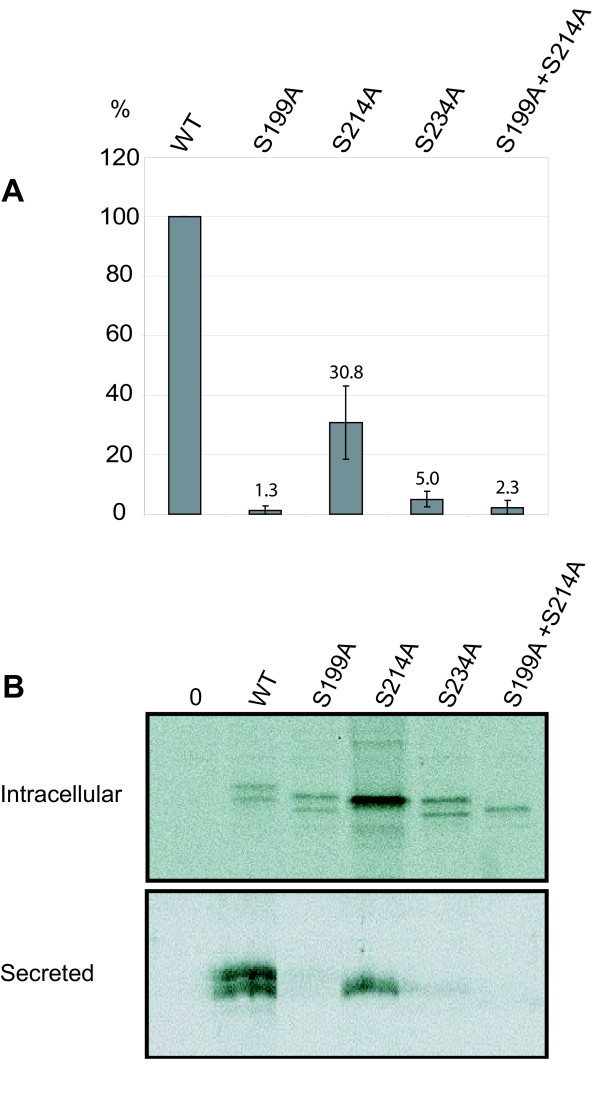
**Activity and processing of wild type and glycosylation site mutated PPT1**. **(A) **COS-1 cells were transfected with plasmids producing mutated PPT1 enzymes and lysed 48 h post-transfection. PPT1 enzyme activity of each lysate (5 μg total protein) was analyzed by a one-hour reaction in + 37°C. Transfection efficacies were controlled by immunofluorescence analysis. The data shown represents an average of three independent experiments and the standard deviations between these experiments are marked as error bars. **(B) **COS-1 cells were transfected and metabolically labelled with 35S-Cysteine for 1 hour, chased for 2 hours, immunoprecipitated, separated in SDS-PAGE and autoradiographed. Both intracellular and secreted forms of PPT are shown.

**Figure 2:**
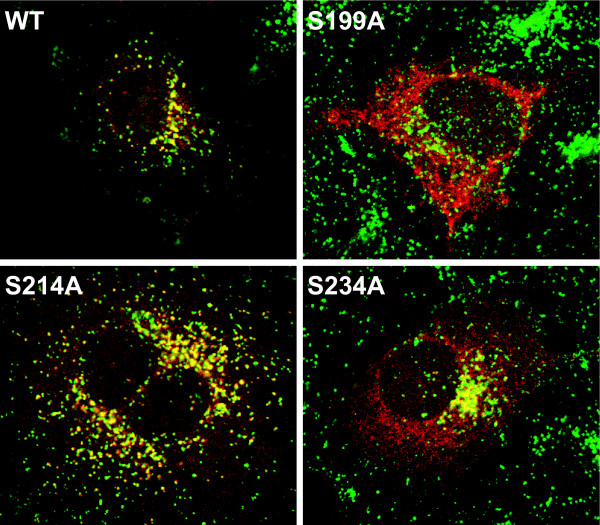
**Intracellular localization of wild type and glycosylation site mutated PPT1 in HeLa cells**. HeLa cells were transfected with the wild type pCMV5-PPT1 plasmid or with plasmids carrying mutations S199A, S214A, S214A and S199A + S214A. The cells were double-stained using the polyclonal antibody for PPT1 (red) and the monoclonal antibody for LAMP-1, lysosomal membrane protein (green). Colocalization is shown in yellow.

PPT1 has three N-glycosylation sites at amino acid positions 197, 212, and 232, and in each of them at least one N-acetylglucosamine residue is present in the crystal structure [19]. Glycosylation results in four forms of overexpressed PPT1 which can be detected as a 32 kDa non-glycosylated form and as 34, 36, and 38 kDa mono-, di- and triglycosylated forms in COS-1 cells [20]. Mutations in glycosylation sites of PPT1 have been shown to affect protein folding and degradation, enzymatic activity, lysosomal sorting, and transport [16]. In our study, we mutagenized the serines to alanines in each PPT1 N-glycosylation consensus site (N-X-S/T) since substitution of serine in the motif supposedly causes only minimal distortion to the polypeptide structure [21]. The mutagenized PPT1 constructs, S199A, S214A, and S234A as well as the double-mutant construct S199A + S214A were transfected into COS-1 and HeLa cells and analyzed for activity, processing, and intracellular localization. The activities of the glycosylation mutants were calculated from cell lysates normalized to transfection efficiency. The results demonstrated that the polypeptide carrying the S214A mutant presented relatively high enzyme activity (31% of normal activity on average), whereas the polypeptides carrying S199A or S234A substitutions showed severely reduced activities, on average 1–5% of normal activity (Figure 1A). The PPT1 polypeptide carrying a double mutation at the glycosylation sites S199A and S214A, also expressed very low enzyme activity. The intracellular processing of the glycosylation mutants was analyzed by metabolic labelling and immunoprecipitation. After a one-hour pulse followed by a 2-hour chase, the mature wild-type PPT1 was detected as a doublet of 36/38 kDa both in the cells and the media (Figure 1B). PPT1 carrying glycosylation site mutations S199A and S234A was detected as doublets and S214A as a major single band, suggesting that both of the remaining glycosylation sites are effectively used in the case of this mutant. The S214A polypeptide was also secreted into the culture media, demonstrating that this protein was able to pass the quality control system of the ER for secretion. PPT1 carrying the S234A mutation was secreted into the culture media in low yields, while S199A and S199A + S214A – mutants were fully non-secretory, suggesting that they mostly retain in the ER. Thus, the current analysis suggests that the N-glycosylation at asparagine 212 is not crucial for PPT1 activity or folding. Earlier studies have shown that PPT1 with a single mutation at any of the three possible glycosylation sites is still capable of obtaining a mannose-6-phosphate (M6P) tag on its sugar structures and binding to M6P-receptor [16]. Das and co-workers have shown that the N232Q mutation in PPT1 has the greatest effect on M6P-receptor binding. Therefore, we also analyzed the intracellular localization of the glycosylation mutated proteins in transfected COS-1 cells by immunofluorescence analysis. As suggested by the enzyme activity measurements, PPT1 carrying the S214A mutation was transported to lysosomes whereas the S199A – mutant was retained in the ER. Mutant S234A showed partial localization to lysosomes, although a major proportion was retained in the ER (Figure 2). The data indicate that the N-glycosylation of asparagines 197 and 232 is more important for PPT1 structure, transport, and function than the N-glycosylation of N212.

### Glycosylation and transport of PPT1 in non-neuronal versus neuronal cells

**Figure 3:**
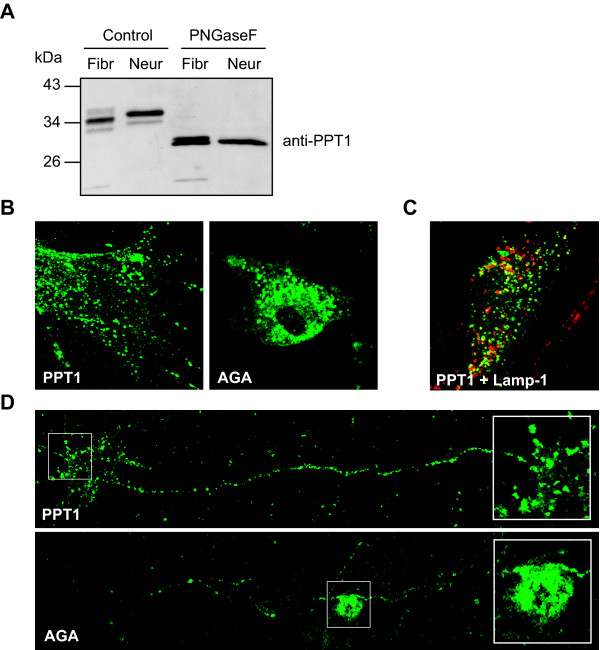
**Adenovirus-mediated PPT1 in fibroblasts and neurons. (A) **Adenovirus-mediated PPT1 was expressed in mouse fibroblasts and neurons. Cell lysates (5 μg of total protein) were treated with PNGaseF to remove the N-glycans from the polypeptides. Proteins were separated with SDS-PAGE and analyzed by immunoblotting. Antibody internalization assay was performed in adenovirus infected mouse fibroblasts **(B) **and neurons **(D) **with both AGA and PPT1 (green). Double staining with the lysosomal marker LAMP-1 (red) is shown for internalized PPT1 in fibroblasts **(C)**. Co-localization is shown in yellow.

Several studies have suggested an extralysosomal localization of PPT1 in neuronal cells [6-8,22], implicating neuron specific properties for PPT1. To approach this question, we overexpressed PPT1 both in mouse fibroblasts and neurons and compared the apparent molecular weights of non-neuronal and neuronal PPT1. Western blot analysis showed that in mouse fibroblasts all three glycosylation forms of PPT1 were present, the diglycosylated form being the most prominent. In neurons, however, only two glycosylation forms could be detected (Figure 3A). Another interesting observation was that PPT1 overexpressed in neuronal cells migrated differently in a SDS-gel than PPT1 produced in mouse fibroblasts. Two neuronal bands focused in the spaces in between the three bands from fibroblasts, rather than at the same level with them. To investigate the reason for this, we treated both samples with PNGaseF to remove all the N-glycans. Repeated Western blot analyses revealed that deglycosylation results in two PPT1 bands in fibroblasts while only one band is seen for neurons (Figure 3A). Also the deglycosylated polypeptides differed in their size, the neuronal band again migrating in between the two bands from fibroblasts rather than at the same level with one of them. This result suggests that in addition to N-glycosylation, PPT1 contains other cell type specific modifications.

We further analyzed the possible disparity in neuronal transport of PPT1 and utilized an antibody internalization assay to compare recycling of PPT1 between non-neuronal and neuronal cells. Mouse fibroblasts and primary neuronal cultures were infected with recombinant adenoviruses encoding PPT1 and a control cDNA, aspartyglucosaminidase (AGA). AGA is a well characterized, classic lysosomal enzyme that has been shown to utilize the M6PR-mediated lysosomal transport and endocytosis also in neurons [23]. As expected, both PPT1 and AGA antibodies were internalized in adenovirus-infected mouse fibroblasts. However, the distribution pattern of the internalized antibody was different. Both AGA and PPT1 localized in vesicular structures, but while AGA localized to perinuclear vesicles, PPT1 was scattered throughout the cell in smaller structures (Figure 3B). AGA showed colocalization with the lysosomal marker Lamp-1 (data not shown), while only a small fraction of PPT1 was localized to lysosomes (Figure 3C). When the same experiment was repeated in neurons, again a clear difference between PPT1 and AGA antibody internalization was observed. PPT1 staining was similar to that seen in the fibroblasts. Small vesicles could be found everywhere in the cell, although the staining was particularly strong in the axons. AGA staining was again strongest in the cell soma, typical of its late endosomal/lysosomal localization [23] and only a few AGA-positive vesicles could be seen in the neuronal projections (Figure 3D). This observation suggests that the transport of recycled PPT1 from the plasma membrane back inside the cell differs from that of AGA in both neuronal and non-neuronal cells.

### Self-oligomerization of PPT1

**Figure 4:**
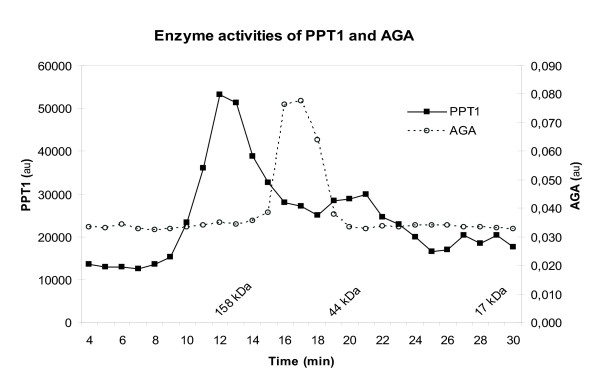
**Size-exclusion chromatography of fractionated PC12 cells: enzyme activities of PPT1 and AGA**. PC12 cells were homogenized and the 10 000g pellet of the postnuclear supernatant was isolated by centrifugation. Solubilised fraction was subjected to size exclusion chromatography and the PPT1 and AGA enzyme activities were measured from the fractions collected. The X-axis indicates time and the molecular weights are derived from the standard (Bio-Rad).

Although the crystal structure of the PPT1 molecule is established, very little is currently known about the biological interactions and the molecular structural format of PPT1 *in vivo*. Therefore, we used Superose 6HR size-exclusion chromatography on cell lysates to determine whether the intracellular PPT1 resides in a monomeric or a complex form. As PPT1 has been categorized as a lysosomal enzyme, we used a subcellular lysosome-containing fraction of PC12 cells as the PPT1 source. The undifferentiated PC12 cells were selected because their endogenous PPT1 levels are high enough for reliable activity measurements. Cell samples were filtrated and fractionated by size-exclusion chromatography. Enzyme activities of both PPT1 and AGA were measured from the elution fractions. AGA participates in glycoprotein breakdown by catalyzing the cleavage of the N-glycosidic bond between asparagine and N-acetylglucosamine [24]. Under native conditions, AGA exists as a dimer (~ 80 kDa) [25]. AGA activity was eluted in a fraction corresponding with the molecular size of a native AGA homodimer (Figure 4). Interestingly, the activity of PPT1 distributed more broadly between the elution fractions. A considerable amount of the PPT1 enzyme activity was demonstrated in the fractions representing apparent MW > 100 kDa. Another PPT1 activity peak was observed in fractions that most probably represent the monomeric form (36–38 kDa) of the enzyme (Figure 4). This result indicates that a significant amount of native PPT1 resides in a complex rather than in a monomeric form.

**Figure 5:**
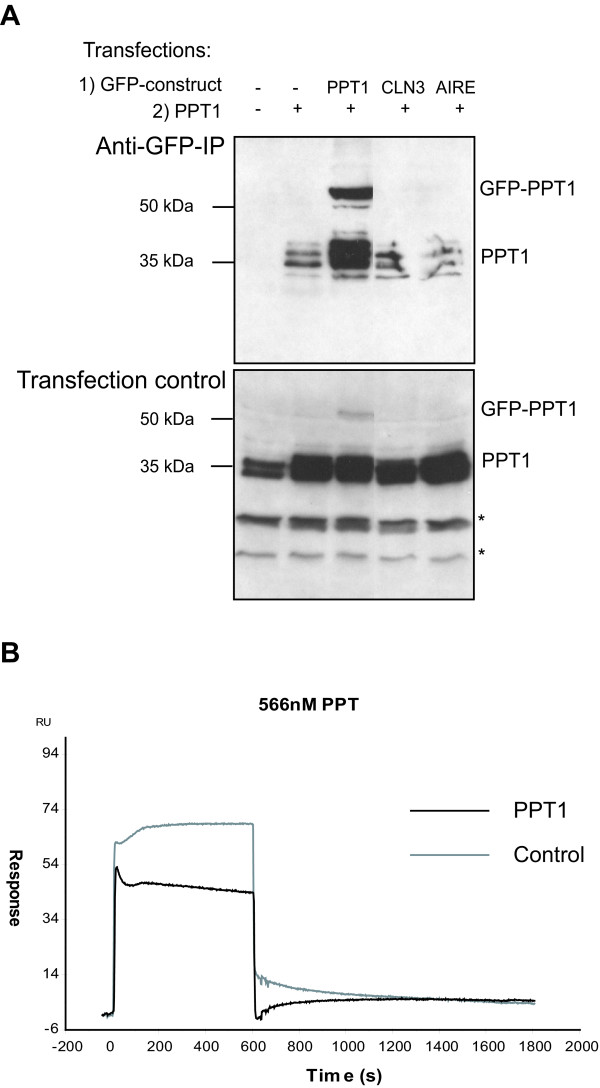
**Oligomerization of PPT1**. **(A) **Cells were transfected with wild type pCMV5-PPT1 plasmids or/and with plasmids producing a GFP-fusion protein as indicated (GFP-PPT1, GFP-CLN3 or GFP-AIRE). The lysates were immunoprecipitated with anti-GFP-conjugated agarose beads. Both lysates (Transfection control) and immunoprecipitated samples (Anti-GFP-IP) were analyzed by immunoblotting using the anti-PPT1 antibody. * = Unspecific binding. **(B) **Biacore assay investigating the self-interaction of purified PPT1 (see methods).

To further investigate the observed *in vivo *complex formation of PPT1, we analyzed the possible self-oligomerization of PPT1 by using co-immunoprecipitation assays. COS-1 cells were double transfected with both GFP-PPT1 fusion protein and wild type PPT1 constructs. Transfected cells were then lysed and co-immunoprecipitated with anti-GFP-agarose beads. Western blot analysis of the co-immunoprecipitates, detected by PPT1 antibody, showed that both the wild type PPT1 and the GFP-PPT1 were present, indicating that GFP-PPT1 and PPT1 interacted with each other (Figure 5A). As controls, we used GFP-CLN3 and GFP-AIRE fusion proteins [26,27]. CLN3 is another NCL protein behind the juvenile form of the disease, shown to be localized to endosomes and lysosomes [28]. AIRE is a nuclear protein participating in T-cell maturation. PPT1 did not co-precipitate with either of these fusion proteins (Figure 5A).

To distinguish whether the self-interaction of PPT1 molecules was direct or mediated by other factors, we also performed a direct interaction assay for purified PPT1. Recombinant PPT1 was purified from the stably transfected CHO-cells [8] by the method modified from Bellizzi et al [19]. The purified enzyme was then used to analyze direct dimerization of PPT1 in a plasmon resonance energy transfer assay using Biacore application. However, the results of the Biacore analysis showed that purified PPT1 was not capable of interacting with itself under these *in vitro *conditions (Figure 5B) suggesting that other as of yet unknown factors mediate the subcellular complex formation of PPT1.

### Effects of disease mutations on the properties of PPT1

**Figure 6:**
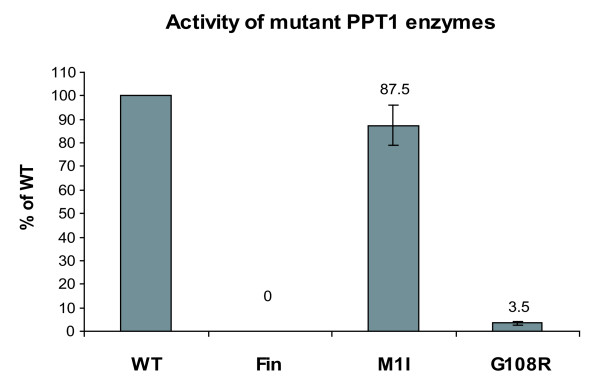
**Enzyme activity of PPT1 with disease mutations in transfected COS-1 cells**. COS-1 cells were transfected with plasmids producing mutated PPT1 enzymes. The cells were lysed 48 h post-transfection and the enzyme activity of each lysate (5 μg of total protein) was analyzed by a one-hour reaction in + 37°C. The data was normalized for PPT1 expression by immunoblotting and densitometric scanning. Here the background activity is assumed to be zero. The data shown represents an average of three independent experiments and the standard deviations between these experiments are marked as error bars.

Mutations in PPT1 have been shown to result in disease with variable age of onset and progression. Previous studies have reported some correlation between the disease mutation phenotype and the localization of PPT1 [17]. Also the effects of known missense mutations on PPT1 activity have previously been explored [16,17,19], but the G108R mutation resulting in an adult phenotype has not been examined in an overexpressing system [29]. Therefore we analyzed the activity of PPT1 carrying G108R and included two other mutants in the analysis, namely M1I (late infantile) and R122W, also denoted as PPT_Fin_, (infantile), the latter as a known inactive control. The activities of the wild type and mutant enzymes were analyzed in transiently transfected COS-1 cells and the data were normalized by immunoblotting and densitometric scanning for the expression level of PPT1 (Figure 6). The initiator codon mutation M1I resulted in the enzyme displaying a nearly full activity but reduced expression. In contrast, the adult onset mutation G108R showed approximately 4% activity compared with that of the wild type enzyme. As a control, PPT_Fin _was lacking the enzyme activity completely.

**Figure 7:**
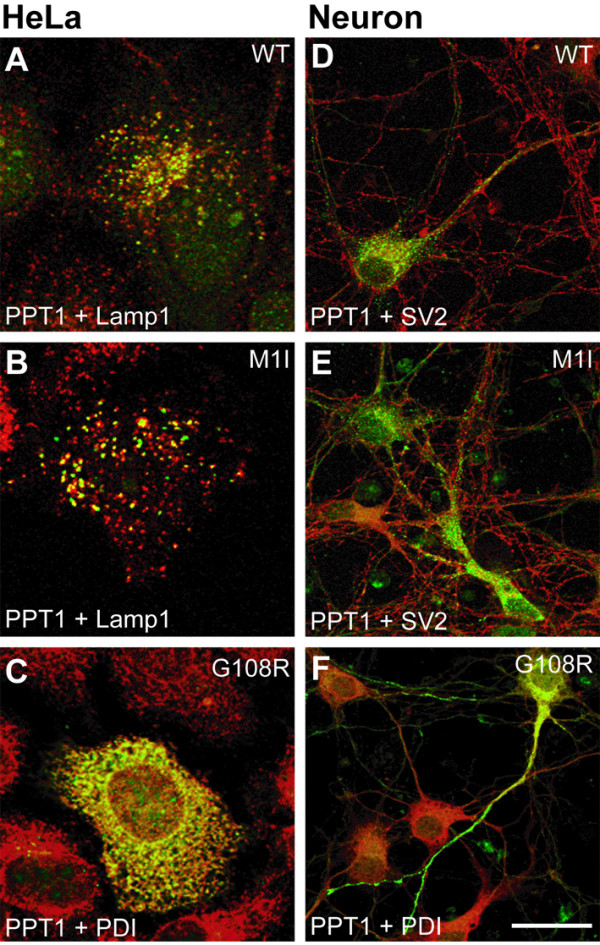
**Intracellular localization of wild type and mutant PPT1 in non-neuronal and neuronal cells**. HeLa cells **(A-C) **transfected with pCMV5-PPT1 plasmids and neurons **(D-F) **infected with PPT1-SFV bearing the indicated mutations were double-stained for PPT1 using the GST-PPT1 antibody (green), for lysosomes using the Lamp-1 antibody (red), for synaptic vesicles using the SV2 antibody (red) and for ER using the PDI antibody (red). Colocalization is shown in yellow. Scale bar = 20 μm.

The subcellular localization of mutated PPT1 has been shown to vary, depending on the cell type and on the type of mutation [4,5,8,17]. We used immunofluorescence staining in transiently transfected HeLa cells and recombinant SFV-infected mouse primary neurons to further map previously unknown intracellular localizations of mutant proteins M1I and G108R. In transfected HeLa cells, double staining together with the late endosomal/lysosomal marker Lamp1 showed that the wild type protein reached the lysosomes, as did the M1I mutant (Figure 7A and 7B). This confirms that the initiator codon mutation M1I (ATG → ATA) results only in a reduced expression level of PPT1 and does not affect the natural intracellular transport of the protein from ER to lysosomes. PPT1 polypeptides carrying the G108R (adult) mutation were retained in the ER and showed a colocalization with the ER marker protein PDI (protein disulfide isomerase) (Figure 7C), similarly to the classical infantile onset R122W mutation (PPT1_Fin_) serving as a control. As in HeLa cells, the distribution of the wild type and M1I enzymes was uniform also in neurons. They localized in the projections of the cells, partially colocalizing with the synaptic vesicle marker SV2 (Figure 7D and 7E). PPT1 with the G108R mutation also displayed some staining in the neuron projections, but did not colocalize with SV2. Instead, it showed partial colocalization with the ER-specific PDI. No overlap with the Golgi marker or the lysosomal marker was detected (data not shown). In conclusion, the G108R mutant causing the adult phenotype does not show significant activity in COS-1 cells and it is not transported similarly to the wild type enzyme in HeLa cells or mouse primary neurons. Thus the activity or the localization was not able to explain the variability in the disease phenotype.

**Figure 8:**
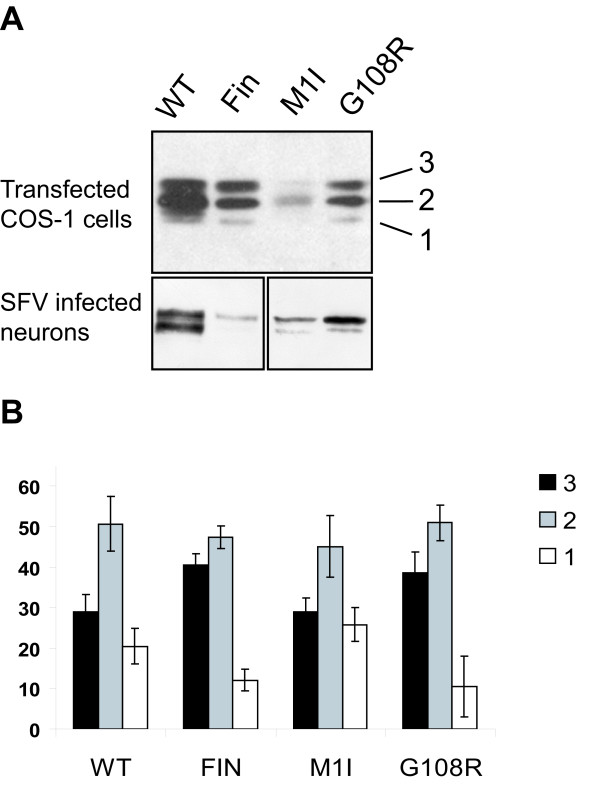
**Glycosylation degree of mutant PPT1 molecules**. **(A) **10 μg of total protein from the cell lysates of transfected COS-1 cells and SFV infected neurons was analyzed by immunoblotting and densitometric scanning. The numbers 3, 2 and 1 indicate the three differently glycosylated forms of PPT1. **(B) **The graph represents relative amounts of tri-, di-, and monoglycosylated forms of PPT1 in transfected COS-1 cells. The data shown represents an average of three independent experiments and the error bars show the standard deviations between these experiments.

Due to the observed difference in the glycosylation degree between neuronal and non-neuronal PPT1, it was of interest to compare the glycosylation degree of the mutant and the wild type proteins in different cell types. We performed an immunoblot analysis from the lysates derived from both transfected COS-1 cells and SFV-infected neurons. The G108R and PPT1_Fin _-mutants harbouring an amino acid substitution in the middle of the polypeptide chain, and thus likely to have disturbances in their folding, presented mostly the tri- and diglycosylated forms of the protein in COS-1 cells (Figure 8A). Notably, no differences could be seen in the glycosylation levels between the mutant proteins causing late or early onset phenotypes. In the case of the PPT1_Fin _and G108R -mutated proteins, the amount of the triglycosylated protein was increased when compared to wild type and M1I-mutated proteins (40% vs. 30%), indicating that in total, the glycosylation degree of the mutants was higher than that of the wild type enzyme or the M1I mutated enzyme (Figure 8B). In neurons, this observation was repeated with even greater differences between the mutants and the wild type protein (Figure 8A). The enzymes with a higher degree of N-glycosylation were the most prominent ones among the mutants, including M1I, unlike in the wild type PPT1, where a lower degree of glycosylation was evident.

**Figure 9:**
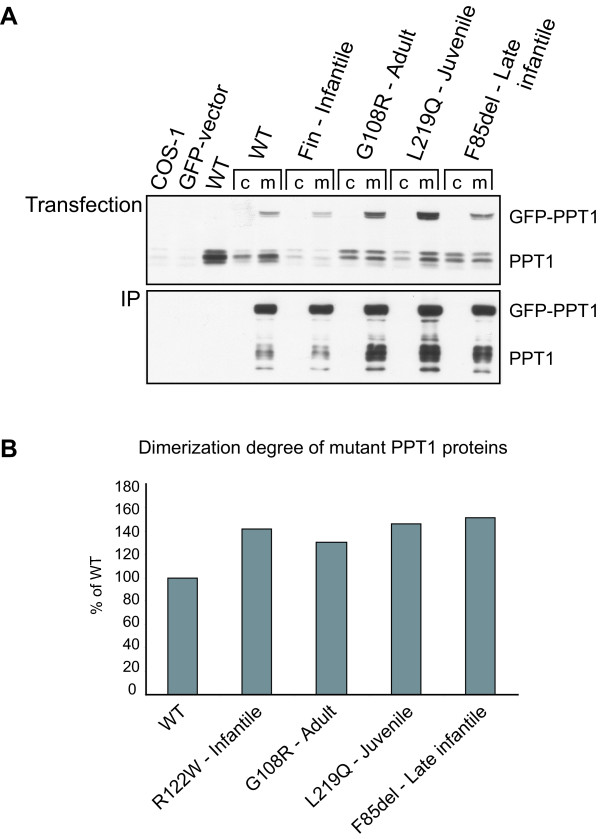
**Oligomerization rate of wild type and mutant PPT1 molecules**. **(A) **COS-1 cells were either single or double transfected with pCMV5-hPPT1 or with plasmids having the indicated mutations, together with GFP-vector (c) or with the corresponding GFP-PPT1 construct having the same mutation in the PPT molecule (m). The cells were lysed 48 h post-transfection. To verify the transfections, 10 μg of total protein from the lysates was analyzed by SDS-PAGE and immunoblotting using both anti-GFP antibody (not shown) and anti-PPT1 antibody (Transfection). The lysates were then subjected to immunoprecipitation using anti-GFP-agarose beads. Immunoprecipitates were analyzed by SDS-PAGE and immunoblotting using the anti-PPT1 antibody (IP). **(B) **The graph represents the dimerization degree of mutant proteins compared to wild type (calculated as a ratio between the immunoprecipitated GFP-PPT/PPT and the transfected GFP-PPT/PPT). An example of a single experiment's quantification is shown experiments.

To investigate the possible role of mutations in the ability of PPT1 to form complexes, four different mutations, each causing a different phenotype, were selected for GFP-pull down analyses: R122W (infantile), F85del (late infantile), L219Q (juvenile), and G108R (adult) (Table 1). To create conditions that mimic a homomeric interaction, we mutagenized both the GFP-PPT1 and PPT1 constructs. After co-immunoprecipitation with GFP-agarose and Western blot detection with PPT1 antibody, we compared the extent of dimerization of the mutated polypeptides with that of the wild type proteins, taking into account the differences in the expression levels (Figure 9A). The experiment was replicated several times, and although we could not quantitate the results repeatedly, the analyses demonstrated that all the mutated PPT1 polypeptides were able to form homodimeric complexes and that the interaction intensities between mutant proteins were somewhat stronger than those observed between wild type proteins (Figure 9B). In summary, our data suggest that the mutated proteins utilize their glycosylation sites more efficiently than the wild type proteins in neurons as well as in other cell types studied here. They also show higher degree of oligomerization than the wild type molecules. However, neither of the described properties can be directly used to explain the variations in the disease onset age.

**Table 1:** Phenotype variability of the CLN1-patients

*Mutation*	*Allele inherited with*	*Phenotype*	*Reference*
	Y109D (c325T > G)	LINCL	
**M1I **(c3G > A)			[42]
	Y247H (c739T > C)	JNCL	
**F85del **(c252-254delCTT)	Exon skipping		
		LINCL	[17]
	(IVS6-1G > T)		
**G108R **(c322G > C)	R151X (c451C > T)	ANCL	[14]
**L219Q **(c656T > A)	R151X (c451C > T)	JNCL	[52]

## Discussion

This study was launched to clarify the effects of glycosylation and complex formation on the properties of PPT1. Previously, the three N-glycosylation sites of PPT1 have been shown to be utilized in non-neuronal cells and their effect on PPT1 activity has been studied by mutagenizing the glycosylation site asparagines to glutamines [19]. Single N → Q mutations were reported to cause only minor effects on PPT1 activity, and double mutations resulted in a minimum of 20% activity. In this study, we utilized a different strategy for generating the glycosylation site mutations. Rather than changing the specific asparagines to another amino acid, we altered the glycosylation site consensus sequence N-X-S/T by mutating serines to alanines. This was done to spare the polypeptide backbone and the side chains from any major distortions [21,30]. Interestingly, our results differed significantly from the previous analyses. The activity of all the glycosylation site mutants was greatly reduced, and only the N212 glycosylation site mutant could retain a considerable enzyme activity. Thus, our data implicates that glycosylation is important for PPT1 activity, and the most important site for it is N197, followed by N232 and N212.

It has been shown that the glycosylation of asparagine 232 has a fundamental role in the M6P-receptor binding and thus it was proposed to have an effect on the proper lysosomal transport of PPT1 in non-neuronal cells [16]. Our studies on the intracellular transport of glycosylation site mutants did not confirm these conclusions. We investigated both intracellular and secreted PPT1 by immunoprecipitation and analyzed the localization of the enzyme by immunofluorescence. We showed that the same glycosylation mutations that affected the PPT1 enzyme activity also had the most severe impact on the transport of PPT1. When the N197 glycosylation site was mutated, PPT1 was retained in the ER, although the N232 glycosylation site – mostly involved in the M6P-receptor binding – was unaffected. When the N232 glycosylation site was mutated, PPT1 localized to the ER – but also partially to lysosomes. The same mutant was also secreted into the media. Thus, our data suggest that the role of M6P-receptor-mediated lysosomal trafficking might not be as crucial for PPT1 as it has previously been thought.

The relative inefficiency of gene therapy trials to treat INCL suggests that the trafficking of PPT1 may have novel properties. M6P-receptor-mediated trafficking has offered a substantial advance to treat lysosomal storage disorders since most soluble lysosomal enzymes can diffuse in the brain tissue. For example, in glucoproteinoses, gene therapy and bone marrow transplantation have been advantageous in animal models and even in human studies, especially in cases where the M6PR-mediated trafficking of the lysosomal enzyme has been shown to operate in neurons [23,31-35]. However, the therapeutic studies in Ppt1-deficient mice involving AAV-mediated PPT1, have ameliorated the CNS pathology only in localized areas, suggesting that the neuronal trafficking of PPT1 has undefined features [36-38]. In this study, we could observe several new properties for neuronal PPT1, including altered modification compared to PPT1 in fibroblasts. Both of the cell types analyzed here were infected with the same recombinant PPT1-adenovirus [7] including the human cDNA, so it is unlikely that our finding would result from different splice variants. One could postulate that the glycosylation is actually similar in these cells, and that the differences seen in the number of the bands would result from some other modification. Further studies are needed to clarify the precise nature of these modifications, but phosphorylation and lipid modifications could be potential study targets. With the antibody internalization assay, trafficking differences could be seen both in fibroblasts and neurons compared with another lysosomal enzyme AGA, which is known to utilize the M6P-receptor. While the AGA antibody was endocytosed into perinuclear lysosomes, the endocytosed PPT1 antibody only partially localized to lysosomes, mostly retaining in small vesicles distributed throughout the cell. We conclude that an increasing amount of evidence pinpoints the existence of an alternative pathway for PPT1 sorting in addition to the M6P-receptor-mediated route.

Molecular interactions of several NCL proteins have been partially resolved. CLN5 has been shown to interact with both CLN2 and CLN3 [39]. CLN6, the protein behind the variant late infantile NCL, was recently shown to form dimers in cross-linkage experiments [40]. However, so far the molecular interactions of PPT1 have not been analyzed in detail. Our data suggest that PPT1 can homodimerize or oligomerize *in vivo*. Both GFP-PPT1 and PPT1 proteins used in the co-immunoprecipitation assay are highly glycosylated. Glycosylation usually prevents aggregation and thus the interaction is not likely to be due to a bias via aggregation of overexpressed protein. More importantly, the demonstration of the active enzyme in the large molecular weight fraction of the cell homogenate during the size-exclusion chromatography supports a true interaction between PPT1 molecules, either directly or via other molecules. PPT1 has been crystallized as a monomer from a secreted protein sample [19]. Neither could we detect oligomerization in a secreted, monomeric sample. Thus it is possible that the intracellular oligomerization of PPT1 involves other, yet unresolved molecules. The understanding of the physiological relevance of the oligomerization for the action of PPT1 also awaits further studies. Interestingly however, we could detect that mutant PPT1 molecules show a higher degree of oligomerization. This may represent a means to regulate the activation and/or transport of the enzyme. For example, acyl protein thioesterase 1 (APT1), a functional relative for PPT1, forms a dimer which has to dissociate before the enzyme displays activity [41]. This suggests that the dimer formation and dissociation may occur reversibly also in the case of PPT1.

PPT1 activities of mutant proteins have been carefully analyzed earlier from transfected COS-1 cell lysates, purified protein samples and patient lymphoblasts [16,42]. While severe INCL-mutations produced inactive enzymes, the mutations causing later onset disease showed low residual activity. However, it has not been possible to draw a straight correlation between the level of residual activity and the age of onset, since the late infantile and juvenile phenotype-causing mutations both possessed activities between 6 and 30% of the wild type enzyme. Also in this study, the overexpressed PPT1 carrying the adult phenotype mutation G108R was shown to have only a low activity of 3.5%. The activities of PPT1_G108R _have earlier been measured in patient fibroblasts and leukocytes and they were similar to those of patients with the early onset disease [14]. As the residual PPT1 enzyme activity of mutant proteins have not correlated with the disease phenotypes, previous studies have proposed that decreased enzyme stability plays a major role in determining the phenotype [16]. A late onset mutation M1I displayed almost full activity (87.5% of wild type) as analyzed by overexpression in COS-1 cells. It has been proposed that the start codon ATA would be utilized in the patients' tissues, resulting in low but clinically relevant expression of the normal enzyme [16]. Our current observations support this suggestion, since also the intracellular localization of this mutant was uniform with wild type enzyme both in HeLa cells and neurons. It can be concluded that the enzyme activity level *per se *cannot be used to distinguish later onset diseases from each other.

In the current study we could not define the molecular basis for the adult phenotype caused by the G108R mutation. The G108R polypeptides fully colocalized with the ER marker PDI in HeLa cells being well in line with earlier findings [17]. Neuronal G108R was transported further to the extensions but did not colocalize with the presynaptic markers, the target for the wild type and M1I polypeptides. Evidently PPT1_G108R _can delay the development of the disease until adulthood without reaching the synaptic vesicles. This may implicate that PPT1 has some functional roles along the secretary pathway. Further research is needed to locate the place(s) of action for neuronal PPT1 and to clarify the molecular networks affected by PPT1 deficiency.

## Conclusion

In this study we show that the N-glycosylation of asparagines 197 and 232 is important for PPT1 activity. We further describe the ability of PPT1 to form complexes. Disease mutations resulting in a classic early onset or later onset forms of INCL were shown to have an effect both on the glycosylation degree of PPT1 and its ability to form complexes, but neither of these phenomena could explain the genotype-phenotype correlation. The current study also demonstrates that intracellular transport and maturation of PPT1 show properties that differ from those involved in the classic mannose 6-phosphate receptor mediated sorting. Prominent differences in the endocytic trafficking of PPT1 can be seen when compared to a well characterized lysosomal hydrolase. Also the size and the modifications of neuronal PPT1 differ from those of PPT1 produced in fibroblasts. Therefore, further investigations in this complex field are essential to resolve the precise disease mechanisms of INCL. Most importantly, clarification of the neuronal transport properties of PPT1 will offer a basis for developing novel therapeutic strategies.

## Methods

### Construction of expression plasmids and recombinant Semliki Forest viruses

The preparation of cDNAs encoding wild type and mutant (F85del, R122W, L219Q) PPT1 inserted in pCMV5 expression vector have been previously described by Salonen et al. [17]. The GFP-PPT1 fusion protein construct was prepared as follows: the EGFP sequence was PCR multiplied with the primers 5'AAAACTGCAGATGGTGAG CAAGGGCGAGGAGCTGT and 3'GTAACCCTGCAGCTTGTACAGCTCGTCCATGC utilizing the pEGEP-C1 plasmid (Clontech) as a template. The resulting PCR fragments were digested with Pst I and ligated to the Pst I site located 3' to the signal sequence of PPT1 at amino acid 26 (His). The mutagenesis of the patient mutations M1I (c3G > A), F85del (c252-254delCTT), G108R (c322G > C), Y109D (c325T > G), R122W (c364A > T), Q177E (c529C > G), V181M (c541G > A) and L219Q (c656T > A), were performed in the PPT1-pCMV5 and GFP-PPT1-pCMV5 using a site-directed mutagenesis kit according to the manufacturer's protocol (Stratagene). The mutagenesis of the three glycosylation sites of PPT1, S199A (c595-596AG > GC), S214A (c640T > C), S234A (c700T > C) and a double mutant of the two first sites S199A + S214A, were performed similarly. The reading frames were confirmed with ABI3730 Automatic DNA Sequencer using the BigDye™ Terminator Cycle Sequencing Kit v3.1 (Applied Biosystems). Recombinant Semliki Forest Virus (SFV) encoding the wild type PPT1 was constructed as previously described [8]. The coding regions of the mutant PPT1 (M1I, G108R, Y109D) were cloned to the *Bam*HI site of the pSFV1 vector and the recombinant PPT1-SFV were prepared as previously described [43].

### Animals

C57/BL WT and C57/BL *Ppt1*^Δex4 ^mice [44] were bred and housed in National Public Health Institute's facilities. The study was approved by the Laboratory Animal Care and Use Committee of the National Public Health Institute, Helsinki. The study has been carried out following good practice in laboratory animal handling and the regulations for handling genetically modified organisms.

### Cell culture, transfections, and SFV infections

COS-1 and HeLa cells were cultured in Dulbecco's modified Eagles' medium (DMEM) supplemented with 10% fetal calf serum, 50 mg/ml streptomycin and 100 IU/ml penicillin, 5% CO_2_/37°C. PC12 cells were cultured on collagen coated dishes in RPMI 1640 medium supplemented with 10% horse serum, 5% fetal calf serum and antibiotics. Mouse hippocampal and cortical neurons as well as mouse fibroblasts were prepared from wild type C57/BL mouse embryos and maintained as previously described [6,45]. For the glycosylation assays and immunofluorescence, COS-1 and HeLa cells were single transfected similarly using mutated (M1I, G108R, Y109D, Q177E, and V181M) and wild type PPT1-pCMV5 constructs. All cells were transfected using the calcium phosphate method as described previously [46]. The cells were used for different experiments 48 h after transfection. For immunofluorescence and glycosylation infections, mouse neurons were cultured for six days *in vitro *(6 div) and infected with recombinant SFV-PPT1 virus (wild type, M1I, G108R, Y109D, and R122W) for 1 h at 5% CO_2_/37°C. The virus was removed and the cells were incubated further for 8 h in the original medium.

### Immunofluorescence staining

For immunofluorescence analysis, the neurons were fixed with 4% paraformaldehyde in PBS for 15 min at room temperature and washed three times with PBS. To increase the penetration of antibodies and to avoid unspecific binding, the cells were incubated in 0.5% BSA and 0.1% Triton X-100 in PBS for 30 min and in 0.5% BSA- PBS for another 30 min. Transfected HeLa cells were fixed and permeabilized with cold methanol for 2 min in -20°C, washed twice with PBS and incubated in 0.5% BSA- PBS for 30 min. In neurons, double immunostainings were performed with rabbit anti-human PPT1 antibody (1:700) together with either mouse ER antibody anti-protein disulfide isomerase (PDI 1:50, Stressgen) or mouse anti-synaptic vesicles (SV-2 1:50) obtained from the Developmental Studies Hybridoma Bank. In HeLa cells, anti-human PPT1 antibody was used with either PDI, Golgi antibody anti-giantin (1:1000) provided by Hans-Peter Hauri (Department of Pharmacology, Biozentrum, University of Basel, Switzerland) [47], or lysosomal membrane glycoprotein antibody anti-human LAMP1 (H4A3, 1:100) from the Developmental Studies Hybridoma Bank (University of Iowa, IA). The cells were incubated with primary antibodies diluted in 0.5% BSA-PBS for 1 h and washed three times with PBS. Secondary antibody incubation was performed with the rhodamine (TRITC)-conjugated anti-mouse IgG + IgM and fluorescein (FITC)-conjugated anti-rabbit IgG (1:250, Jackson ImmunoResearch Laboratories). The coverslips were mounted with Gel Mount (Biomeda Corp.) and viewed with Leica confocal microscopy. The images were further processed with Adobe Photoshop CS and Adobe Illustrator CS software.

### Metabolic labelling and immunoprecipitation

COS-1 cells were cultured in Dulbecco's modified Eagle's medium supplemented with 10% fetal calf serum and antibiotics. For transfection, the cells were seeded on 3 cm plates at a density of 3 × 10^5 ^cells per well. Transfection was performed with the FuGENE 6 transfection reagent (Roche) using 1 μg of the wild-type or mutant *PPT1 *cDNA construct per well. Following a 48 h incubation the cells were metabolically labelled for 1 h with [^35^S]Cys (Amersham Pharmacia Biotech) followed by a 2 h chase. Immunoprecipitation was carried out with fixed *Staphylococcus aureus *cells (Calbiochem) using a GST-PPT antibody (1:700). The polypeptides were denatured by boiling them 5 min prior to loading. The labelled polypeptides were separated by 14% SDS–PAGE under reducing conditions and visualized by autoradiography.

### Adenovirus infection, deglycosylation, and antibody internalization assay

Construction of recombinant adenoviruses Ad-PPT1_wt _and Ad-AGA_wt _and the adenovirus infection protocols have been described previously [7,48]. Mouse primary neurons were grown on cover slips for 8 days before infection, infected for an hour and grown for another four days before the antibody internalization experiment or western blot analysis. Mouse primary fibroblasts were seeded on cover slips one day before infection and the antibody internalization assay and western blot analysis were done 24 h after infection. For antibody internalization assay, cover slips were incubated two hours in medium containing antibodies against PPT1 (8414, 1:700 [6]) or AGA (1:200, [49]). Cover slips were transferred to normal growth media for another 2 hours and fixed with 4% PFA. To visualize the internalized antibody, cells were stained with a secondary antibody. For double immunostaining, LAMP-1 was used as described above. For deglycosylation analysis, the cells were lysed with D'cell Angelica buffer (50 mM Tris-HCl, pH 7.4, 300 mM NaCl, 1% Triton X-100 0.1% BSA) and the lysates were treated with N-glycosidase F (PNGaseF; New England Biolabs) according to the manufacturer's instructions. In short, 5 μl of total protein was treated with 2 μl of PNGaseF in 20 μl reaction buffer for 1–16 h at 37°C. Samples were analyzed with 14% SDS-polyacrylamide gel and immunoblotting using a PPT1 specific polyclonal antibody.

### PPT1 purification and antibodies

Conditioned media from a recombinant CHO-PPT1 cell line [8] containing secreted PPT1 was collected and concentrated with Centriprep YM-10 concentrators (Millipore) adjusted to 5 mM HEPES pH 7.0 and 0.5 M NaCl and applied to Hi Trap Phenyl HP column (Amersham Pharmacia). The column was washed with 5 mM HEPES buffer with decreasing salt concentration and PPT1 was eluted with 5 mM HEPES, 70% ethanol, pH 7.9. To neutralize the sample, it was eluted into tubes containing 1 M HEPES, pH 7.0. The buffer was then changed to 5 mM HEPES, 150 mM NaCl and the sample was applied to a HR-12 column for size-exclusion chromatography. The volume of this further purified sample was reduced with Centricon YM-10 concentrators (Millipore). The final yield was about 1.15 mg of PPT1 per 300 ml of crude medium. Rabbits were immunized by four sequential subcutaneous injections of 160 μg of PPT1 in Freund's complete adjuvant. The blood was collected two weeks after the last immunization and the serum was further affinity purified using GST-PPT1 fusion protein. The antibody titers and specificity were determined by immunofluorescence, Western blot, and immunohistochemistry.

### Surface plasmon resonance analysis

Binding of PPT1 with itself was studied with surface plasmon resonance analysis in a Biacore 2000 biosensor (Biacore). A saturating amount of purified PPT1 was covalently attached to a flow cell of a biosensor chip using standard amine coupling according to the manufacturer's protocol. Varying concentrations of PPT1 was diluted in Hepes buffered saline (HBS) with two different pH-values (5 and 7.4) and used as analytes. An empty flow cell was used as a negative control.

### PC12 cell fractionation and size-exclusion chromatography

PC12 cells were homogenized in HB-buffer (3 mM imidazole, 250 mM sucrose, pH 7.4) with a glass homogenizer. The homogenate was centrifuged at 800 *g *for 10 min in an eppendorf tube centrifuge to provide the PNS. To provide the lysosomal fraction, the supernatant was further centrifuged at 10 000 *g *for 20 min. The pellet was washed with HB-buffer and recentrifuged at 10 000 *g *for 20 min. The pellet was resuspended in D'ell Angelica buffer (see above) and filtrated with 0.22 μm Spin-X filter tubes (Costar). Size-exclusion chromatography of the sample was performed with a Superdex 75 column (*V *= 25 ml, Amersham Pharmacia Biotech) in PBS at room temperature. The flow rate was 0.25 ml/min and fractions of 0.25 ml were collected. Molecular weight markers for the size-exclusion chromatography were purchased from BioRad Laboratories.

### Enzyme activity assays and glycosylation degree analysis

The aspartylglucosaminidase (AGA) activity assay was based on the colorimetric measurement of liberated N-acetylglucosamine from the synthetic substrate 2-acetamido-1-β-(L-aspartamido)-1,2-dideoxy-β-D-glucose (AADG) as described earlier [50]. For PPT1 enzyme activity and Western blot analysis, transfected COS-1 cells and SFV-infected mouse primary hippocampal neurons were washed twice with cold PBS and lysed with freeze-thaw-cycles in water supplemented with protease inhibitors (Complete, Roche). The PPT1 activity assay was performed as described earlier [51] using 5 μg of total protein from crude cell lysate or 30 μl from size-exclusion chromatography fractions. The substrate (4-methylumbelliferyl-6-thiopalmitoyl β-D-glucoside, 4-MU-6S-Palm-βGlc) was purchased from Moscerdam Substrates. The amount of released 4-MU was determined with a fluorometer. Free 4-MU (Sigma) diluted in stop-buffer (0.5 M NaHCO_3_/Na_2_CO_3 _pH 10.7, 0.025% Triton X-100) was used as a standard. For glycosylation degree analysis, 10 μg of total protein was assayed with 14% SDS-PAGE and immunoblotting using a PPT1 specific polyclonal antibody. This antibody was raised in rabbit against recombinant PPT1 produced by the baculovirus expression system (code 8414) [6]. The specificity of the antibody was tested by western blot using purified PPT1 and both preimmune and immune rabbit serums for the detection (data not shown). The protein bands were visualized by ECL (Amersham Biosciences) or alkaline phosphatase reaction (New England Biolabs) and quantitated by densitometric scanning using Scion Image software (Scion Corporation).

### GFP-coimmunoprecipitation and immunoblotting

For the GFP-coimmunoprecipitation assays, COS-1 cells were seeded one day before transfection to 92 mm dishes and grown to 50–80% confluency. The cells were double transfected with mutant PPT1-pCMV5 and GFP-PPT1-pCMV5 (wild type, R122W, G108R, L219Q, and F85del) constructs. Single transfections using pEGFP-C1 vector, PPT1-pCMV5 and Herring Sperm DNA were used as controls. Cells were collected 48 h after transfection and lysed with D'ell Angelica buffer (see above). A sample of the crude lysate was taken for transfection analysis and the rest was immunoprecipitated with anti-GFP agarose beads (GFP (B-2) AC: sc-9996 AC, Santa Cruz Biotechnology). Samples were analyzed with 14% SDS-polyacrylamide gel and immunoblotting using a PPT1 specific polyclonal antibody.

## Authors' contributions

AL carried out the protein purification and complex formation experiments as well as the experiments concerning the disease mutations, participated in the antibody internalization assay and drafted the manuscript. CvS carried out the experiments with the glycosylation mutants and helped to draft the manuscript. TS participated in the study design and molecular cloning of the disease mutation constructs. OK participated in the neuronal cell culture management and designed the assays concerning the glycosylation mutants. JS participated in the activity assays and the design of glycosylation mutants. MJ participated in the study design concerning the complex formation. AK participated in the study design of the antibody internalization assay and in drafting the manuscript. AJ and TS conceived of the study, and AJ participated in its design, coordination and drafting the manuscript. All authors read and approved the final manuscript.
